# Gastric Necrosis in a Previously Healthy Child: A Case Report

**DOI:** 10.34172/mejdd.2024.392

**Published:** 2024-07-31

**Authors:** Shahnam Askarpour, Hazhir Javaherizadeh, Mahboobeh Rashidi, Mahmood Khoshkhabar, Afshin Rezazadeh

**Affiliations:** ^1^Department of Pediatric Surgery, Children’s Medical Center, Ahvaz Jundishapur University of Medical Sciences, Ahvaz, Iran; ^2^Alimentary Tract Research Center, Clinical Sciences Research Institute, Imam Khomeini Hospital, Ahvaz Jundishapur University of Medical Sciences, Ahvaz, Iran; ^3^Department of Anesthesiology and Intensive Care Unit, Children’s Medical Center, Ahvaz Jundishapur University of Medical Sciences, Ahvaz, Iran; ^4^Department of Pediatric Radiology, Children’s Medical Center, Ahvaz Jundishapur University of Medical Sciences, Ahvaz, Iran

**Keywords:** Case report, Corona virus, Gastric, Necrosis

## Abstract

Gastric necrosis is a very rare surgical emergency in a previously healthy child. A 13-year-old boy with abdominal pain and coffee-ground vomiting was admitted to the emergency department. Physical examination revealed signs of peritonitis and septic shock. The patient underwent a laparotomy. Gastric necrosis and discoloration of the lower esophagus and duodenum due to ischemia were present. Distention of gastric and duodenum was also seen. Total gastrectomy and Roux-en-Y esophagojejunostomy were done. The patient underwent a chest computed tomography (CT), and patchy ground-glass opacity was observed in both lungs. Consolidation was seen in the lower lobe of the lung. The polymerase chain reaction (PCR) for coronavirus was tested two times. The first time was negative, and the second time was positive. The patient was discharged in good condition. During the follow-up period, severe anastomotic strictures occurred. In our case, gastric necrosis and positive coronavirus were reported.

## Introduction

 Gastric necrosis and perforation in a healthy child without previous medical and surgical history have been reported.^1^ Gastric necrosis may be due to excessive gastric dilatation caused by a high volume of carbonated beverages or meals.^1^ This condition was seen in a patient with an eating disorder such as anorexia nervosa or bulimia.^2,3^ Coronavirus has some presentation in children, including pulmonary or gastrointestinal presentation.^4^ Vomiting, diarrhea, and abdominal/gastric pain were the most common presentations of coronavirus among patients.^5^ To date, gastric necrosis associated with the pulmonary manifestation of coronavirus has not been reported in the literature. In this case report, we describe a child with gastric necrosis and followed perforation, pulmonary involvement, and fever with positive polymerase chain reaction (PCR) of coronavirus.

## Case Report

 A 13-year-old boy with acute abdominal pain and vomiting was admitted to the emergency department. Inability for defecation was reported. Physical examination revealed severe distention, generalized tenderness, and symptoms of septic shock. A nasogastric tube was fixed. The coffee ground material was observed in the nasogastric tube. The patient had a 40° C fever. Laboratory examinations during first hospital admission were as the following: White blood cell (WBC) = 11.4, red blood cell (RBC) = 4.50, hemoglobin = 9 g/dL, Na = 138 mmol/L. Arterial blood gas showed acidosis (pH = 7.04, Hco3 = 17.04 mEq/L, Pco2 = 63.5 mm Hg). Gastric volvulus was excluded using abdominal imaging.

 The patient underwent a laparotomy in the hospital. Severe dilatation was seen in the two-thirds proximal of the stomach and duodenum. The distal pulse of limbs was weak. The patient was confused. A surgical incision was made in the first hospital. Due to severe illness, an incision was closed after irrigation, and the patient was referred to a referral center.

 At the referral center, a physical examination revealed abdominal distension and abdominal guarding. A nasogastric (NG) tube was fixed. Fecaloid material was seen in the NG tube aspirate. The second laparotomy was done in the referral hospital.

 One liter of bloody liquid was seen in the gastric space. Severe dilatation was seen in two-thirds proximal to the stomach. Dilatation was seen in the duodenum. A pulse of the superior and inferior mesenteric arteries was detected. Generalized ileus and distension of the bowel loop due to peritonitis were noted. White blood cell count during admission was 4.9 × 10^3^/µL, 5.58, 3.1, 5.3, and 9, respectively.

**Figure 1:**
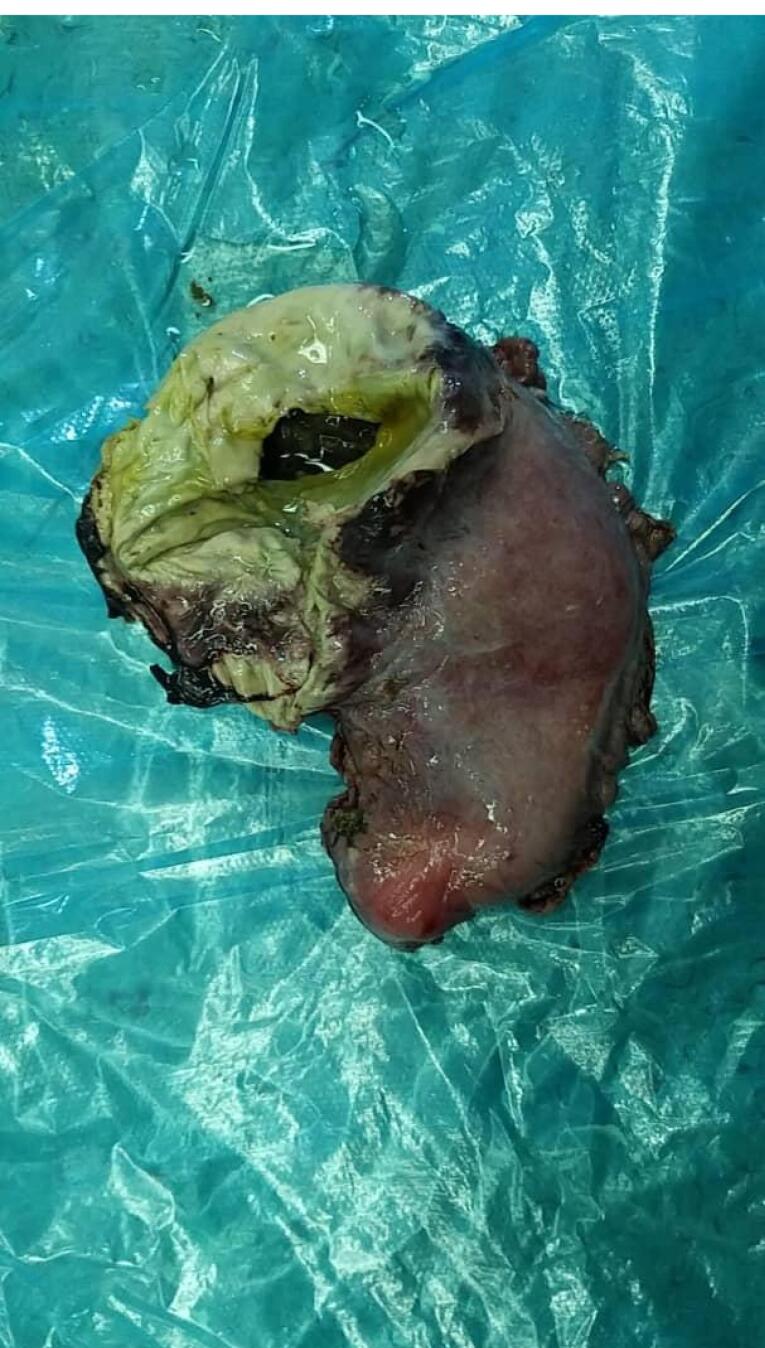
Necrotic stomach

**Figure 2:**
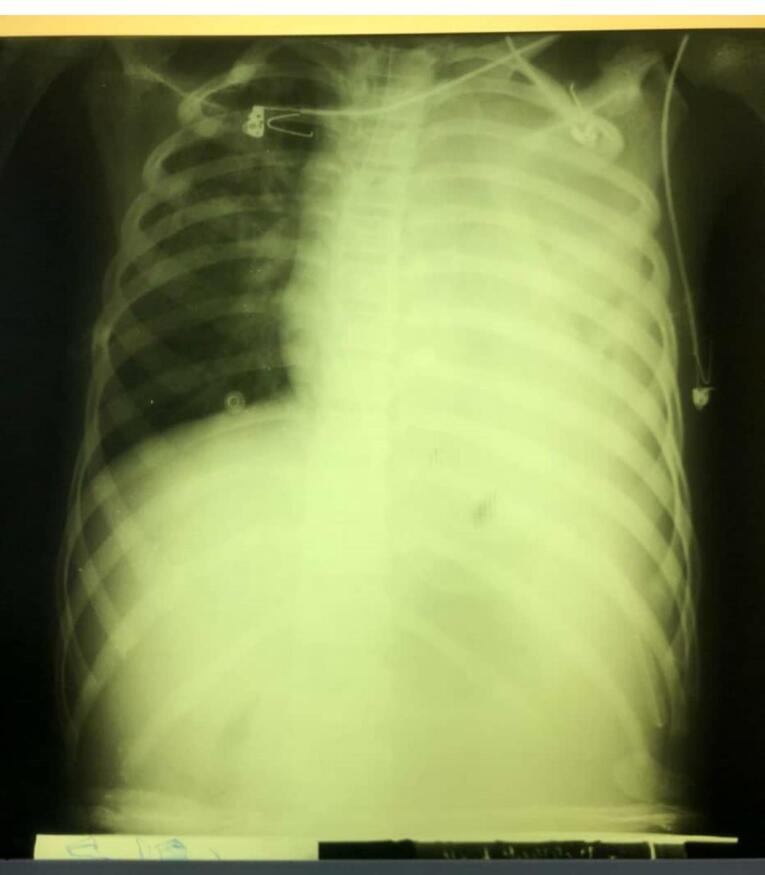
Pleural effusion was seen in the left hemithorax

**Figure 3:**
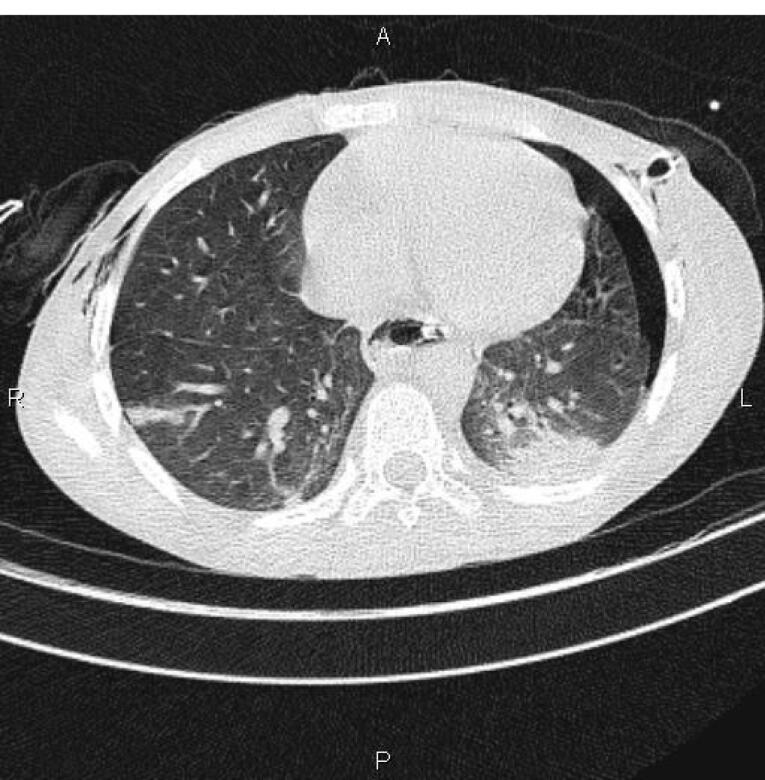
Patchy ground glass opacity was seen in bilateral lungs

 Total gastric necrosis was seen (Figure 1). Total gastrectomy and Roux-en-Y esophagojejunostomy were done. Duodenum was not involved. Other abdominal organs appeared normal. Due to pleural effusion, which was seen in the chest radiograph (Figure 2), the chest tube was inserted. Coronavirus 19 PCR was requested. PCR was done for the child. The first report was negative, and the 2^nd^ time was positive. A Chest computed tomography (CT) scan was requested. Patchy ground-glass opacity was seen in both lungs (Figure 3). Consolidation was seen in the lower lobe of the lung. The patient was discharged after intensive care unit (ICU) admission. The patient was visited 2 weeks later after discharge and was in good condition. During the follow-up period, the patient showed feeding problems and anastomotic site stricture. The patient was treated using electrocautery surgery with a good outcome.^6^

## Discussion

 Acute gastric necrosis and rupture were rare events in children. Acute gastric perforation was reported following a large volume of carbonated beverages.^1^ Acute gastric perforation was also seen in patients with anorexia or bulimia nervosa disorder.^3^ Cardiovascular problems such as Kawasaki among children infected with coronavirus 2019 were reported in the literature.^7^ Vascular problems may involve gastric mucosa. Perforation of intestinal mucosa following Kawasaki was reported in the literature.^8^

 Pleural effusion was reported among COVID-19 patients.^9^ In our case, left-sided pleural effusion was seen.

 In the study by Ma and colleagues on 50 children with positive PCR of COVID-19, 14% had no evidence of disease on chest CT.^10^ Ground glass opacity was the most frequently reported pattern, and it was seen in 67% of chest CT scan findings.^10^

 A low white blood cell count was seen in our case. Low WBC count was reported in the literature among patients affected by severe acute respiratory syndrome (SARS) coronavirus.^10^

 Although we described a child with pulmonary involvement and gastric necrosis, the cause-and-effect relationship between coronavirus and gastric necrosis in a child without previous medical or surgical background should be proved with future research.

 Gastric necrosis is a rare condition among children. We evaluated our coronavirus case, but the result of PCR was negative in the 1^st^ evaluation and positive in the 2^nd^ evaluation. Negative polymerase chain reactions for COVID-19 cases were reported in the literature.^11,12^

## Conclusion

 Gastric necrosis is a very rare event in children. This report may be the first report of the cooccurrence of coronavirus and gastric necrosis in a child. However, there is an absence of evidence of the cause-and-effect of coronavirus infection and gastric necrosis. The association of coronavirus and gastric necrosis may be an incidental finding in our cases.
